# A conductive and thixotropic dual-network hydrogel based on *Alyssum campestre* seed gum and *Matricaria chamomilla* L. extract

**DOI:** 10.1039/d6ra05964c

**Published:** 2026-08-12

**Authors:** Mona Baniasadi, Mahsa Baghban Salehi, Hossein Baniasadi

**Affiliations:** a Department of Chemical Engineering, Chemistry and Chemical Engineering Research Center of Iran Tehran Iran m.bsalehi@ccerci.ac.ir mahsa.bsalehi@gmail.com; b Polymer Synthesis Technology, School of Chemical Engineering, Aalto University Espoo Finland hossein.baniasadi@aalto.fi

## Abstract

Developing conductive hydrogels with balanced mechanical, electrical, and biological properties remains a major challenge for biomedical applications. In this study, a multifunctional conductive dual-network hydrogel was developed using *Alyssum campestre* seed gum (ACSG) and *Matricaria chamomilla* L. extract (CE). The hydrogel network was constructed by combining a chemically crosslinked polyacrylamide network with Mg^2+^-mediated physical interactions to form an interpenetrating dual-network structure. FTIR analysis indicated the incorporation of CE into the hydrogel matrix and suggested possible intermolecular interactions, while SEM observations revealed that CE produced a more compact porous microstructure with reduced pore size. Thermogravimetric analysis showed that incorporating CE did not adversely affect the hydrogel's thermal stability. The CE-containing hydrogel exhibited improved viscoelastic properties, including a higher storage modulus, an extended linear viscoelastic region, and rapid self-recovery during cyclic strain measurements. In addition, the CE-containing hydrogel exhibited ionic conductivities of (4.99 ± 0.78) × 10^−3^ and (7.09 ± 1.60) × 10^−5^ S cm^−1^ in the freshly prepared and equilibrium-swollen (ESR) states, respectively. Furthermore, the hydrogels maintained high H9c2 cell metabolic activity (>90%) after 72 h. These findings demonstrate an effective strategy for tuning the structural, rheological, electrical, and biological properties of ACSG-based conductive dual-network hydrogels.

## Abstract 2

Developing conductive hydrogels with balanced mechanical, electrical, and biological properties remains challenging for biomedical applications.

## Graphical abstract

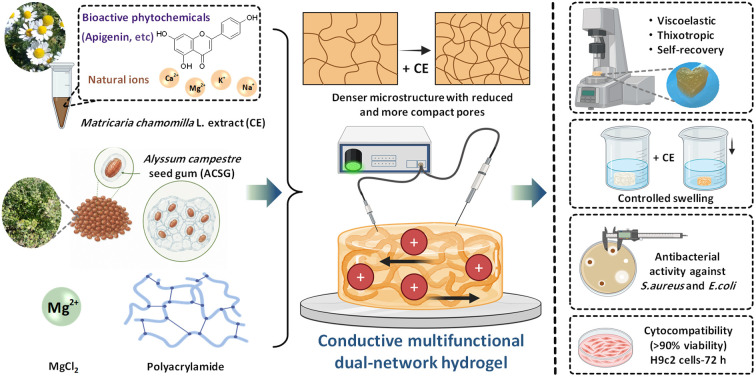

## 1. Introduction

Hydrogels have attracted considerable attention as biomaterials for tissue engineering because of their highly hydrated structure, soft tissue-like characteristics, and ability to mimic the three-dimensional (3D) extracellular matrix (ECM).^1–4^ However, for electroactive tissues such as cardiac muscle, skeletal muscle, and neural tissues, mechanical support alone is insufficient to restore or maintain physiological function.^5,6^ Consequently, conductive hydrogels have emerged as promising candidates for engineering electroactive tissues. To date, various conductive nanomaterials and intrinsically conductive polymers have been incorporated into hydrogel systems to improve electrical performance,^7^ including MXene-based cardiac patches, nanofiller-reinforced scaffolds, and polypyrrole-containing hydrogels, all of which have demonstrated enhanced electromechanical properties and biological performance.^8–12^

Unlike electronically conductive materials, ionic-conductive hydrogels transport electrical signals through the migration of ions, closely resembling the conduction mechanism of native biological tissues. Recent advances in biomimetic ion-transport systems, such as artificial potassium channels with dynamic ion selectivity, have further highlighted the importance of controlling ionic migration for developing intelligent bioinspired materials.^13^ Zhou *et al.*^14^ suggested that this mechanism makes ionic-conductive hydrogels particularly suitable for mimicking the physiological behavior of soft tissues. Despite these advantages, the development of sustainable ionic-conductive hydrogels based on natural polysaccharides and plant-derived multifunctional additives remains relatively limited. In particular, strategies capable of simultaneously improving network organization, mechanical stability, conductivity, and biological performance are still needed.

Besides material composition, the architecture of the polymer network plays a decisive role in determining hydrogel performance.^15,16^ Dual-network hydrogels have emerged as an effective approach to overcome the limited mechanical strength of conventional hydrogels by combining a permanently crosslinked network with reversible physical interactions.^17–19^ Such architectures improve toughness, energy dissipation, structural stability, and recovery after deformation compared with a single-network system.^20,21^ For example, Wu *et al.*^22^ demonstrated that introducing dynamic hydrogen bonding into a silk fibroin/acrylamide/graphene oxide/calcium dual-network hydrogel significantly enhanced its mechanical performance. Among the various reversible interactions, ionic crosslinking has proven particularly attractive because it provides dynamic physical junctions while preserving network flexibility and self-recovery behavior.^20,23^

Natural polysaccharides have been extensively explored as hydrogel-forming materials owing to their biodegradability, biocompatibility, abundance, and versatile physicochemical properties.^24,25^ Among them, *Alyssum* seed gum is a plant-derived polysaccharide capable of forming highly hydrated 3D networks.^26^ Its carboxylate-rich structure provides excellent hydration capacity and viscosity,^27,28^ making ACSG a promising platform for functional hydrogel design. Nevertheless, ACSG-based hydrogels still require improved mechanical robustness and electrical functionality for advanced biomedical applications. Previous studies have shown that Mg^2+^ ions promote intermolecular associations between *Alyssum* gum chains and substantially modify their rheological behavior,^29^ suggesting that Mg^2+^-mediated ionic interactions can serve as effective dynamic crosslinking points for constructing mechanically reinforced dual-network hydrogels.

Incorporating multifunctional natural additives represents another attractive strategy for tailoring hydrogel performance. In our previous study, TiC-reinforced ACSG-based semi-interpenetrating polymer network hydrogels exhibited enhanced conductivity and viscoelastic properties, demonstrating the suitability of ACSG as a platform for electroactive biomaterials.^26^ More recently, we showed that *Artemisia ciniformis* extract could act as a natural ionic-conductive component while simultaneously improving the multifunctional properties of hydrogel systems.^30^ However, the role of plant-derived extracts in influencing the physicochemical characteristics of conductive hydrogel networks beyond conductivity remains insufficiently understood. In particular, it remains unclear whether the synergistic contribution of inorganic ions and polyphenolic compounds in plant extracts can influence not only ionic conductivity but also network-related properties, such as swelling behavior, viscoelasticity, and structural organization. Among medicinal plants, *Matricaria chamomilla* L. has received considerable attention due to its high levels of flavonoids and phenolic compounds. *Chamomile* extract (CE) has been incorporated into a variety of biomedical materials owing to its well-established antioxidant, anti-inflammatory, and antimicrobial activities.^31–33^ Nevertheless, previous studies have mainly focused on its biological effects, whereas its influence on hydrogel network formation and the resulting structure–property relationships has received much less attention. The presence of naturally occurring inorganic ions together with hydroxyl- and phenolic-rich compounds suggests that CE has the potential to influence the physicochemical characteristics of hydrogel systems. Such constituents may contribute to changes in network organization and consequently affect swelling behavior, viscoelastic response, ionic conductivity, and biological performance. Despite growing interest in conductive hydrogels, dual-network architectures, and plant-derived bioactive additives, the combined influence of CE and Mg^2+^-mediated dual-network formation on conductive polysaccharide hydrogels has not yet been systematically investigated.

Therefore, in this study, a conductive ACSG-based dual-network hydrogel was developed by integrating a chemically crosslinked polyacrylamide network with Mg^2+^-mediated ionic interactions and CE as a multifunctional natural modifier. Beyond evaluating the conductive role of CE, this work specifically investigates whether a plant-derived extract containing both inorganic ions and polyphenolic constituents can influence the physicochemical characteristics of a dual-network hydrogel system. The effects of CE on network architecture, swelling behavior, viscoelastic properties, ionic conductivity, thermal stability, antibacterial activity, and *in vitro* cytocompatibility were systematically evaluated. Particular emphasis was placed on establishing the structure–property relationships governing the multifunctional performance of the developed hydrogel system.

## 2. Experimental

### 2.1. Materials

Flowers of *Matricaria chamomilla* L. and seeds of *Alyssum campestre* were collected from their natural environments in the Isfahan province, Iran, at elevations of 1600 and 1200 m above sea level, respectively. Acrylamide (AM) served as the main component for the hydrogel framework. To establish the crosslinked configuration, *N*,*N*′-methylenebisacrylamide (MBA) and magnesium chloride (MgCl_2_) were used as agents for covalent and ionic crosslinking, respectively. The polymerization process was initiated using ammonium persulfate (APS). All chemical substances were of analytical grade and obtained from Merck (Germany) without further purification.

### 2.2. Preparation of *Matricaria chamomilla* L. extract

**Fig. 1:**
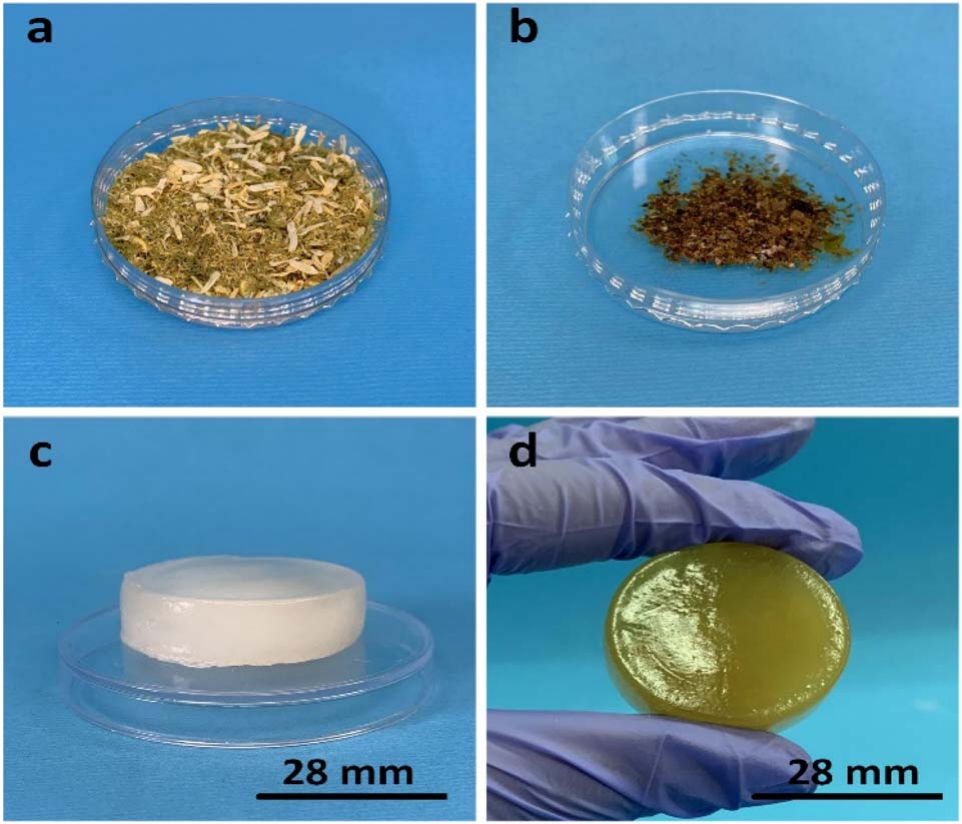
Schematic overview of materials and hydrogel preparation. (a–d) Digital photographs showing milled *Matricaria chamomilla* L., CE, and the fabricated A-M-M-A-H and A-M-M-A-CE-H hydrogels.

To prepare the CE, 20 g of the plant flowers were first pulverized using a laboratory mill. The ground material was then suspended in 100 mL of distilled water and mixed at 80 °C for 15 min. The extracted material was filtered and centrifuged at a rate of 2700 rpm for 20 min. The supernatant was separated, and the residue was freeze-dried to obtain a powdered extract. The final product was stored in a sealed, light-proof container in a refrigerator at 4 °C. Fig. 1a and b shows the digital photographs of milled *Matricaria chamomilla* L. and its final extract.

### 2.3. Synthesis of hydrogel

ACSG was extracted according to our previously reported procedure.^26^ Conductive dual-network hydrogels were prepared by combining ACSG and acrylamide (AM) at a weight ratio of 1 : 3 in distilled water. ACSG (3 wt%) was first hydrated at 45 °C for 30 min, followed by the addition of AM (23 wt%) to obtain a homogeneous precursor solution. The mixture was subsequently deoxygenated under nitrogen while the temperature was gradually increased to 55 °C. CE (10 wt% relative to the total polymer/monomer content) and MgCl_2_ (1 wt%) were then incorporated as multifunctional additive and ionic crosslinking agent, respectively. Covalent network formation was achieved by introducing *N*,*N*′-methylenebisacrylamide (MBA, 4 wt% of AM) as the crosslinker, followed by initiation of free-radical polymerization using ammonium persulfate (APS, 10 wt% of AM) at 70 °C. The resulting hydrogels were allowed to mature at room temperature for 24 h and subsequently purified by sequential washing in acetone and distilled water to remove residual unreacted species. To investigate the effect of CE on network architecture and hydrogel performance, two formulations were prepared, comprising a CE-free control dual-network hydrogel (A-M-M-A-H) and a CE-containing dual-network hydrogel (A-M-M-A-CE-H). Representative photographs of the synthesized hydrogels are shown in Fig. 1c and d.

### 2.4. Characterization

The structure of the produced hydrogel and the intermolecular interactions of its functional groups were analyzed using a Fourier-transform infrared (FTIR) Nicolet 470 spectrometer over a wavenumber range of 4000–500 cm^−1^. Inductively coupled plasma optical emission spectrometry (ICP-OES; Spectro Arcos) was employed to determine the concentration of metal ions in the CE. Energy dispersive X-ray spectroscopy (EDS), attached to a TESCAN Vega3 instrument (Czech Republic), was used to identify and quantify the elemental composition of the hydrogel. Subsequently, the morphology and surface characteristics of the dried hydrogels were investigated using scanning electron microscopy (SEM) after gold coating of the samples. Thermogravimetric analysis (TGA) was performed to assess the thermal behavior of the fabricated hydrogels using a Netzsch TG 209 F1 analyzer (Germany). The measurements were carried out in a nitrogen atmosphere over the temperature range 23–500 °C at a heating rate of 10 °C min^−1^, with changes in sample mass continuously monitored.

The swelling behavior of the hydrogels was evaluated in distilled water at 25 °C by measuring the swelling ratio (SR) and equilibrium swelling ratio (ESR), calculated using eqn (1).^34^1
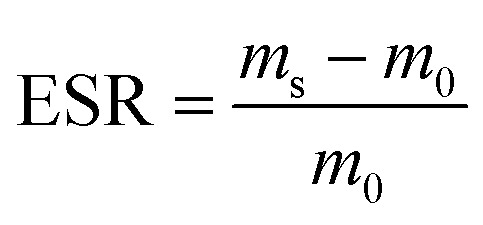
In which, *m*_s_ and *m*_0_ are the weights of swollen and dried hydrogel samples, respectively.

The EC of the hydrogels was evaluated using a qualitative LED-based conductivity tester (Kimyagaran Javan Co., Iran) to confirm their conductive behavior. For quantitative estimation, the resistance of hydrogel samples was measured using a digital multimeter (VC97, Victor, China) under direct contact configuration. The EC (*σ*, S cm^−1^) was calculated based on eqn (2).^30^2
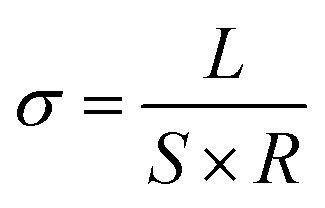
where *L* denotes the distance between the electrodes, *S* represents the area of the sample, and *R* is the measured resistance of the hydrogel.

Rheological measurements were conducted to evaluate the viscoelastic properties and deformation behavior of the hydrogels.^35^ Oscillatory rheological tests were performed using an MCR 300 rheometer (Anton Paar, Austria) equipped with a parallel-plate geometry (plate diameter: 25 mm, gap size: 2 mm). Before each measurement, the hydrogel samples were carefully loaded onto the measuring plate, and excess material was trimmed to ensure proper geometry filling. All tests were conducted at 37 °C to simulate physiological conditions.

First, strain sweep measurements were performed at a constant frequency of 1 Hz over a strain range of 0.01–100% to determine the linear viscoelastic region (LVR) of the samples. The LVR was identified as the strain range over which the storage modulus (*G*′) and loss modulus (*G*″) remained nearly constant, indicating that the hydrogel structure was not disrupted. Next, frequency-sweep tests were conducted within the LVR to investigate the frequency-dependent viscoelastic response of the hydrogels. The variation of *G*′ and *G*″ with frequency was used to examine the stability of the hydrogel network under dynamic conditions. To further evaluate the structural resilience of the hydrogels, step-strain measurements were also performed by alternating between low and high strain levels. This analysis was intended to examine the recovery behavior of the network after a large deformation.

The antibacterial activity of the hydrogels was evaluated using the agar disk diffusion method according to a previously reported protocol.^36^*Escherichia coli* (*E. coli*, ATCC 25922) and *Staphylococcus aureus* (*S. aureus*, ATCC 25923) were selected as representative Gram-negative and Gram-positive bacteria, respectively. Bacterial suspensions at approximately 10^6^ CFU mL^−1^ were spread onto Mueller–Hinton agar plates and incubated for 24 h. Disk-shaped hydrogel specimens (7 mm in diameter) were sterilized under UV irradiation for 20 min on each side and then carefully placed on the inoculated agar surfaces. The plates were subsequently incubated at 37 °C for 12 h. Commercial tetracycline discs (TE30, 30 µg disc^−1^) served as the positive control. Antibacterial activity was assessed by measuring the diameter of the inhibition zones formed around the samples.

The cytocompatibility of the hydrogels was assessed *via* an MTT assay using H9c2 rat cardiac cells as a preliminary *in vitro* model. The assay was performed according to our previously reported method.^26^ Briefly, H9c2 cells were seeded onto sterilized hydrogel samples (1 × 10^4^ cells per well), and cell viability was evaluated after 1, 2, and 3 days using the MTT assay. Absorbance was measured at 550 nm, and cell viability was expressed relative to untreated control cells.

## 3. Results and discussion

### 3.1. Structural characterization of the hydrogels

**Fig. 2:**
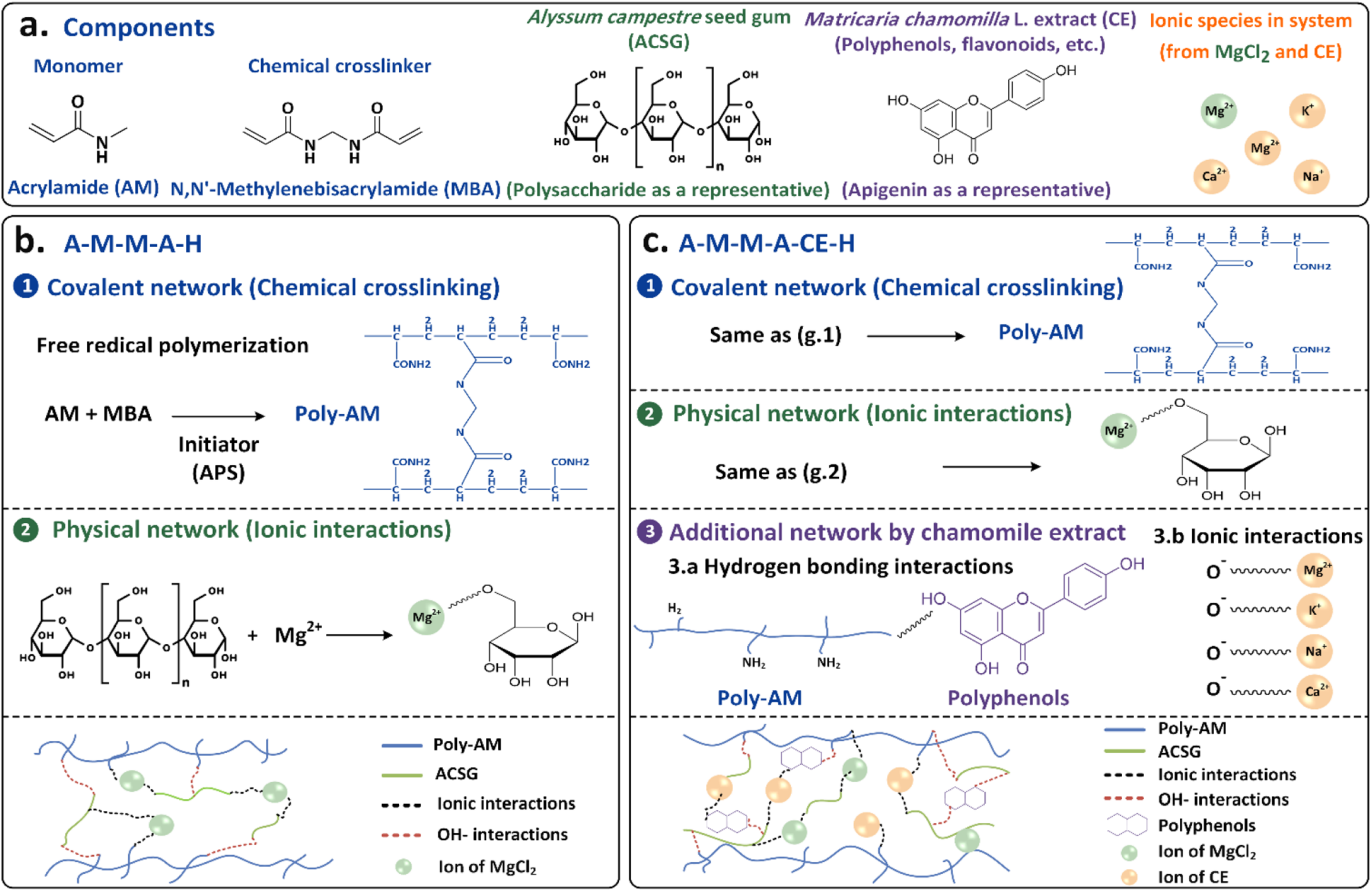
Schematic illustration of the proposed structural features and possible interactions within the hydrogel systems. (a) Components used in the hydrogel system, (b) proposed structure and interactions in the A-M-M-A-H hydrogel, and (c) proposed structure and possible interactions in the A-M-M-A-CE-H hydrogel.

The design strategy for the developed hydrogel system is based on constructing a multifunctional dual-network architecture. In this system, the covalently crosslinked polyacrylamide (poly-AM) network provides structural integrity, while Mg^2+^-mediated interactions with the ACSG chains introduce reversible physical associations within the network. In addition, the incorporation of CE introduces naturally occurring inorganic ions and polyphenolic constituents, which may contribute to additional intermolecular interactions and modify the hydrogel's physicochemical characteristics. The synergistic contributions of hydrogen-bonding interactions, ionic associations, and possible secondary interactions are expected to influence network organization and, consequently, properties such as swelling behavior, viscoelastic response, and ionic conductivity. The proposed structural features and possible interactions within the hydrogel network are schematically illustrated in Fig. 2.

**Fig. 3:**
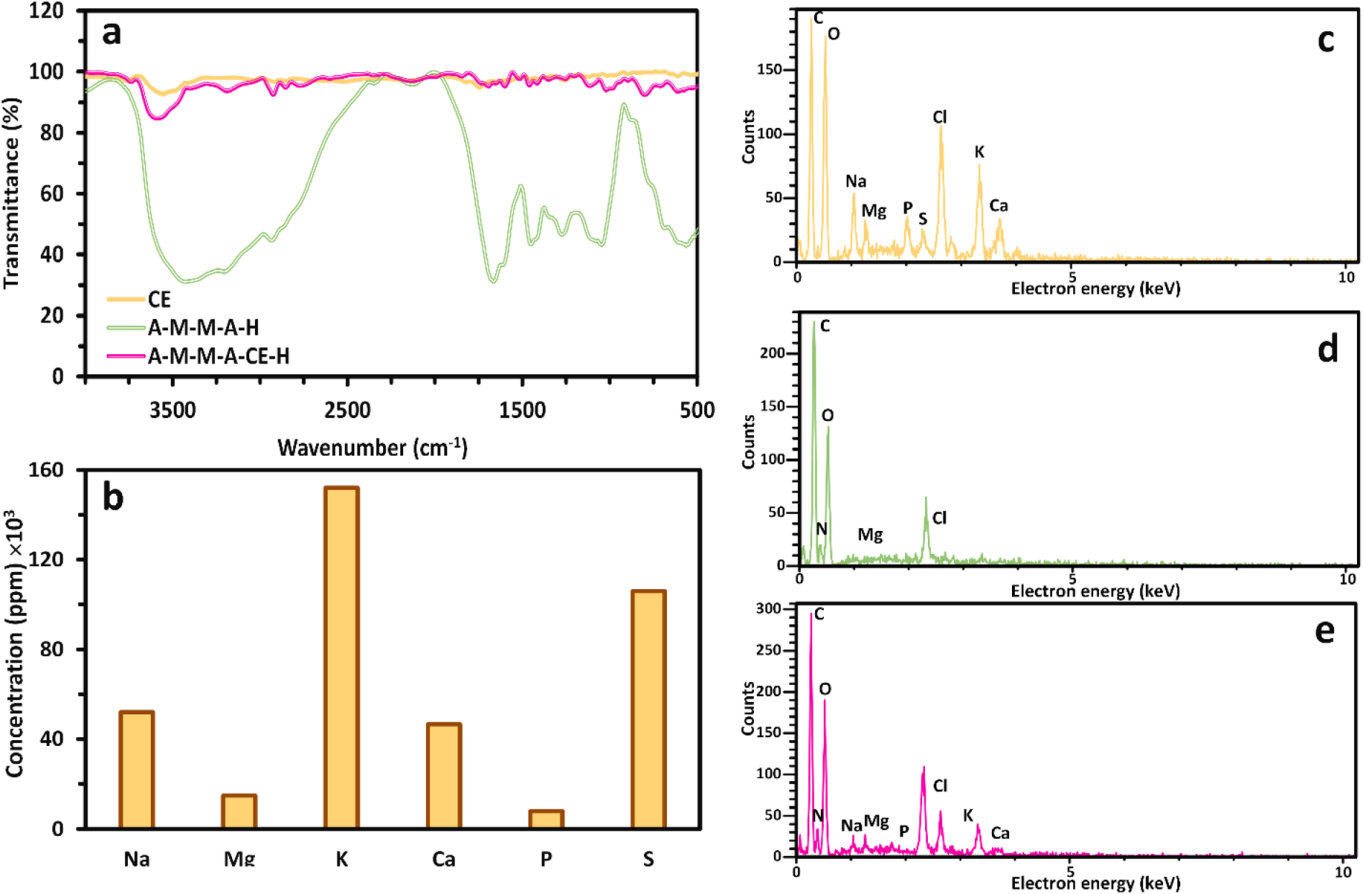
Structural characterization of the hydrogels. (a) FTIR spectra of CE, A-M-M-A-H, and A-M-M-A-CE-H, (b) ICP-OES result of CE, (c) EDS results of CE, (d) EDS results of A-M-M-A-H, and (e) EDS results of A-M-M-A-CE-H.

FTIR spectroscopy was used to evaluate the functional groups of CE and the resulting hydrogels (Fig. 3a). The CE spectrum displayed bands at 3557 and 3410 cm^−1^, corresponding to O–H stretching vibrations of phenolic compounds and flavonoids.^32,33,37^ Absorption bands between 1697 and 1651 cm^−1^ were also assigned to carbonyl-containing and aromatic constituents of CE.^32^ The absorption band observed at approximately 1720 cm^−1^ was attributed to C=O, which may originate from carbonyl-containing compounds such as matricin present in *Matricaria chamomilla* L. Moreover, apigenin, as one of the bioactive flavonoids in *chamomile* extract, contains carbonyl groups in its chemical structure that may contribute to this absorption region.^32^

For A-M-M-A-H, the broad band at 3433 cm^−1^ was attributed to the overlapping O–H and N–H stretching of ACSG and poly-AM.^26^ It has been reported that Mg^2+^ ions can interact with oxygen-containing functional groups in similar polysaccharide systems, which may contribute to slight variations in this region.^38^ The peaks at 1666 and 1606 cm^−1^ were assigned to C=O stretching and NH_2_ vibration of the poly-AM network, respectively, while the bands at 1454 and 1280 cm^−1^ corresponded to CH_2_ bending and C–N stretching vibrations.^39^ The band at 1049 cm^−1^ was related to the C–O and C–O–C vibrations of the polysaccharide backbone.^26^

In the A-M-M-A-CE-H sample, several spectral changes were observed. Hydroxyl-related bands appeared at 3588, 3402, and 3186 cm^−1^,^40^ and slight shifts occurred in the amide region compared to the control hydrogel.^41^ The observed shifts and intensity variations suggest the possible occurrence of secondary intermolecular interactions, including hydrogen bonding, between the phenolic/hydroxyl groups of CE and the amide/hydroxyl groups of the hydrogel components.

Additionally, the absorption band at 1411 cm^−1^ may be attributed to enhanced hydrogen-bonding interactions associated with the polyphenolic constituents of CE.^17^ Changes in the fingerprint region (1111 and 1018 cm^−1^) also suggest overlapping vibrations of C–O, C–O–C, and phenolic groups.^42^ The FTIR spectra showed characteristic functional groups associated with the hydrogel components and CE, while the observed spectral variations suggested possible interactions between CE and the hydrogel matrix.

To identify the inorganic constituents of the CE, its elemental composition was analyzed by ICP and EDS. As shown in Fig. 3b and c, the presence of Na, K, Ca, Mg, P, and S was detected, indicating that CE contains a range of naturally occurring inorganic ions derived from the plant matrix. EDS analysis of the A-M-M-A-CE-H hydrogel (Fig. 3d and e), in comparison with the CE-free sample (A-M-M-A-H), further confirmed the incorporation of these elements into the hydrogel system. Although some of these ions may originate from physically entrapped extract components, their presence within the hydrogel matrix suggests their potential involvement in the network's ionic microenvironment.

The presence of multivalent ions, particularly Mg^2+^ and Ca^2+^, may facilitate ionic associations with oxygen-containing groups of ACSG, including carboxylate groups, thereby potentially influencing the dynamic characteristics of the network. These possible interactions, together with the covalent poly-AM framework and secondary interactions associated with CE constituents, may contribute to the observed changes in swelling behavior, viscoelastic response, and ionic conductivity.

### 3.2. Microstructural analysis and pore morphology

**Fig. 4:**
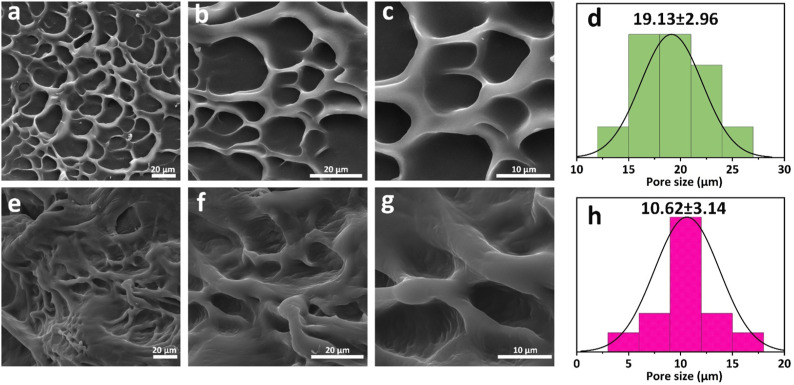
Microstructural characterization of the hydrogels. SEM micrographs of the control sample (A-M-M-A-H) at (a) 1.3 k×, (b) 3 k×, (c) 6 k× magnification, (d) pore size distribution histogram for A-M-M-A-H. SEM micrographs of A-M-M-A-CE-H sample containing the extract at (e) 1.3 k×, (f) 3 k×, (g) 6 k× magnification, and (h) pore size distribution histogram for A-M-M-A-CE-H.

The internal architecture of the synthesized hydrogels was investigated using SEM, as porosity and pore connectivity are key parameters that influence mass transport and cellular interactions in hydrogel systems. As shown in Fig. 4a–c, the control A-M-M-A-H hydrogel exhibited a well-defined 3D macroporous structure with highly interconnected and uniformly distributed pores, with an average pore size of 19.13 ± 2.96 µm (Fig. 4a and d). Such interconnected porosity is essential for facilitating nutrient diffusion and waste removal,^43,44^ which are critical for maintaining cellular activity in tissue-engineered constructs.^26^

Following the incorporation of CE, notable changes in the internal morphology were observed. The A-M-M-A-CE-H hydrogel exhibited a denser microstructure with smaller, more compact pores (Fig. 4e–g). Quantitative analysis revealed a significant decrease in average pore size to 10.62 ± 3.14 µm (Fig. 4e and h). This reduction, together with apparent thickening of pore walls, suggests that CE may influence the network formation process and lead to a more compact polymer architecture. This morphological evolution may be associated with additional secondary interactions within the hydrogel matrix. Based on ICP and EDS results, the presence of inorganic ions and polyphenolic constituents in CE may increase ionic and hydrogen-bonding interactions with the polymer chains. These interactions may act as supplementary physical crosslinking points, restricting polymer chain mobility and resulting in a denser network structure. Importantly, despite this densification, the hydrogel maintains a macroporous architecture suitable for mass transport and cellular interactions. This structural variation may influence mechanical properties, which are further discussed in the rheological analysis.

### 3.3. Thermal degradation behavior

**Fig. 5:**
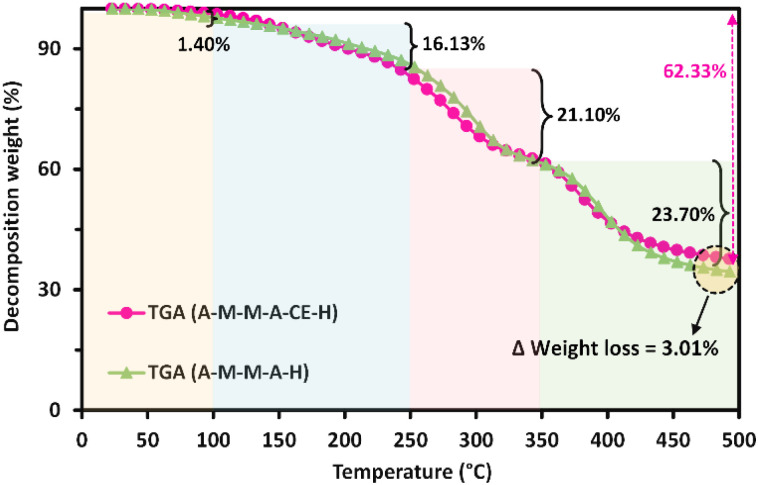
TGA of A-M-M-A-H and A-M-M-A-CE-H recorded from 25 to 500 °C under a nitrogen atmosphere.

The thermal stability and degradation behavior of the A-M-M-A-H and A-M-M-A-CE-H hydrogels were investigated using TGA, as shown in Fig. 5. Both samples exhibited a typical multi-stage degradation profile characteristic of polysaccharide–polyacrylamide-based hybrid networks. An initial minor weight loss of approximately 1.40% below 100 °C was attributed to the removal of physically adsorbed water from the hydrophilic matrix.^45,46^

The main degradation occurred between 100 and 350 °C, corresponding to the decomposition of polysaccharide segments and polyacrylamide chains.^47^ At higher temperatures (>350 °C), further breakdown of the polymer backbone led to the formation of a carbonaceous residue.^48,49^ The incorporation of CE resulted in a slight improvement in thermal stability, as reflected by a lower total weight loss at 500 °C for A-M-M-A-CE-H (62.33%) compared to the control hydrogel. This modest enhancement suggests that CE does not disrupt the primary network structure and may contribute to additional intermolecular interactions within the hydrogel matrix, as also supported by FTIR and SEM analyses. The results indicated that the incorporation of CE maintains the thermal integrity of the hydrogel system without compromising its structural stability.

### 3.4. Swelling behavior and ionic conductivity

**Fig. 6:**
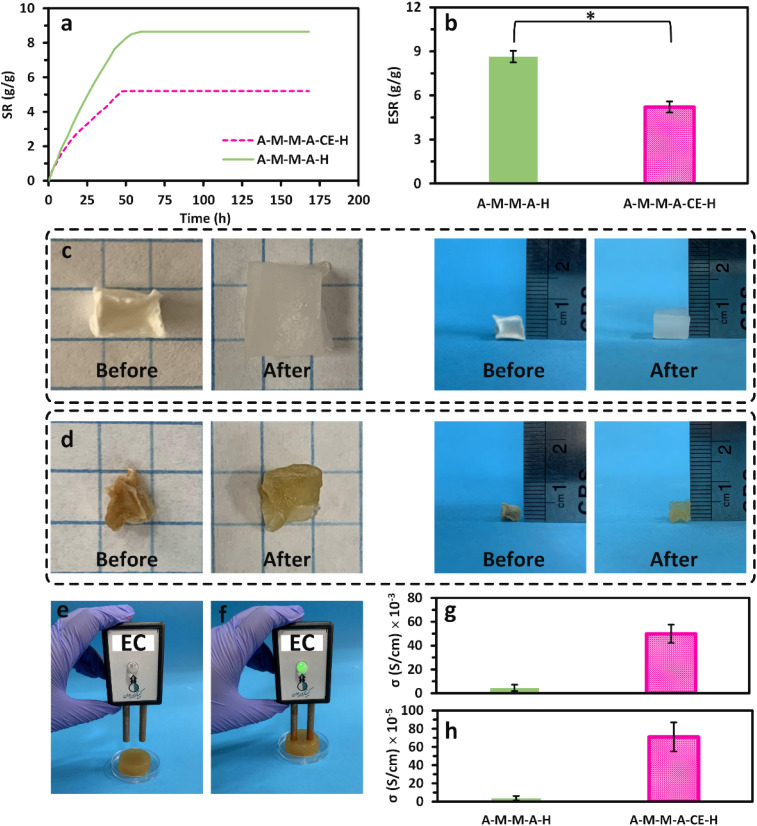
Swelling and electrical characterization of the hydrogels. (a) Swelling kinetics, (b) ESR of A-M-M-A-H and A-M-M-A-CE-H (*n* = 3, mean ± SD; *P* < 0.005, unpaired two-tailed *t*-test), (c) photographs of A-M-M-A-H in dry and ESR states, (d) photographs of A-M-M-A-CE-H in dry and ESR states, (e and f) qualitative EC demonstration using an LED-based tester, (g) quantitative EC comparison between A-M-M-A-H and A-M-M-A-CE-H in the freshly prepared state, and (h) EC comparison between A-M-M-A-H and A-M-M-A-CE-H at ESR.

Controlled swelling plays an important role in determining the performance of hydrogel-based systems,^50^ as it directly influences mass transport, structural stability, and ion mobility within the network. Excessive swelling may lead to dilution of the polymer network, resulting in weakened structural integrity and reduced ion transport efficiency.^14^ The swelling behavior of the synthesized hydrogels is shown in Fig. 6a and b, while digital images of the dry and ESR are presented in Fig. 6c and d. The results revealed that the A-M-M-A-H hydrogel exhibited an ESR of 8.64 ± 0.39 after 52 h. In contrast, the incorporation of CE significantly reduced water uptake, with the A-M-M-A-CE-H sample reaching an ESR of 5.20 ± 0.75 after 60 h. This 1.66-fold decrease in swelling was found to be statistically significant (*P* < 0.005).

The reduced swelling behavior of the CE-containing hydrogel is consistent with the microstructural observations (Fig. 4), in which a denser, more compact network structure was identified. This structural evolution suggested that the presence of CE influences network formation, likely through additional physical interactions. Specifically, phenolic compounds and inorganic ions present in the extract may contribute to the formation of supplementary hydrogen bonding and ionic interactions with the polymer chains. These interactions can increase the effective crosslinking density and restrict polymer chain expansion, thereby limiting water diffusion into the hydrogel matrix. Such controlled swelling behavior is beneficial for maintaining the hydrogel's structural integrity under hydrated conditions, preventing excessive network expansion while preserving a stable porous architecture suitable for subsequent ion transport and functional performance analysis.

The EC of the hydrogels was investigated to evaluate the effect of CE on ion-transport properties within the polymer network (Fig. 6e–h). Initially, a qualitative assessment was performed using LED- and buzzer-based circuits. As demonstrated in Fig. 6e and f, the A-M-M-A-CE-H sample successfully completed the electrical loop, resulting in LED illumination and buzzer activation, thereby confirming the ionic conductivity of the hydrogel. Quantitatively, incorporating CE resulted in higher conductivity. In the as-prepared state, A-M-M-A-CE-H exhibited a conductivity of (4.99 ± 0.78) × 10^−3^ S cm^−1^, compared with (4.44 ± 2.67) × 10^−4^ S cm^−1^ for the control hydrogel (Table S1). These results are consistent with the contribution of mobile ionic species to charge transport, potentially arising from the inorganic ions present in CE together with Mg^2+^ ions within the hydrogel matrix.

After reaching ESR, the A-M-M-A-CE-H hydrogel maintained a higher conductivity ((7.09 ± 1.60) × 10^−5^ S cm^−1^) compared with the control sample ((3.65 ± 2.45) × 10^−6^ S cm^−1^), indicating that ionic transport pathways were partially retained after swelling. The decrease in conductivity after ESR compared with the as-prepared state is attributed to water uptake-induced network expansion and dilution of mobile ionic species, which reduces the effective concentration of charge carriers within the hydrogel matrix. Nevertheless, the presence of hydrated polymer networks and ion-containing components allows continued ion migration. It is worth noting that conductivity measurements in the swollen state were performed in distilled water. In contrast, studies performed in electrolyte-containing media such as PBS typically report higher conductivity values due to additional contributions from external ions.^51^ Therefore, the present results provide a conservative estimation of the intrinsic ionic conductivity of the hydrogels. Despite this limitation, the A-M-M-A-CE-H hydrogel exhibited conductivity values within the range reported for ionically conductive hydrogel systems,^52,53^ confirming the effective role of CE in enhancing ion transport within the network. The conductivity values were comparable to those reported for ionically conductive hydrogel systems,^30^ indicating the potential of the developed network for applications requiring ion-transport-mediated electrical functionality.

### 3.5. Rheological behavior

**Fig. 7:**
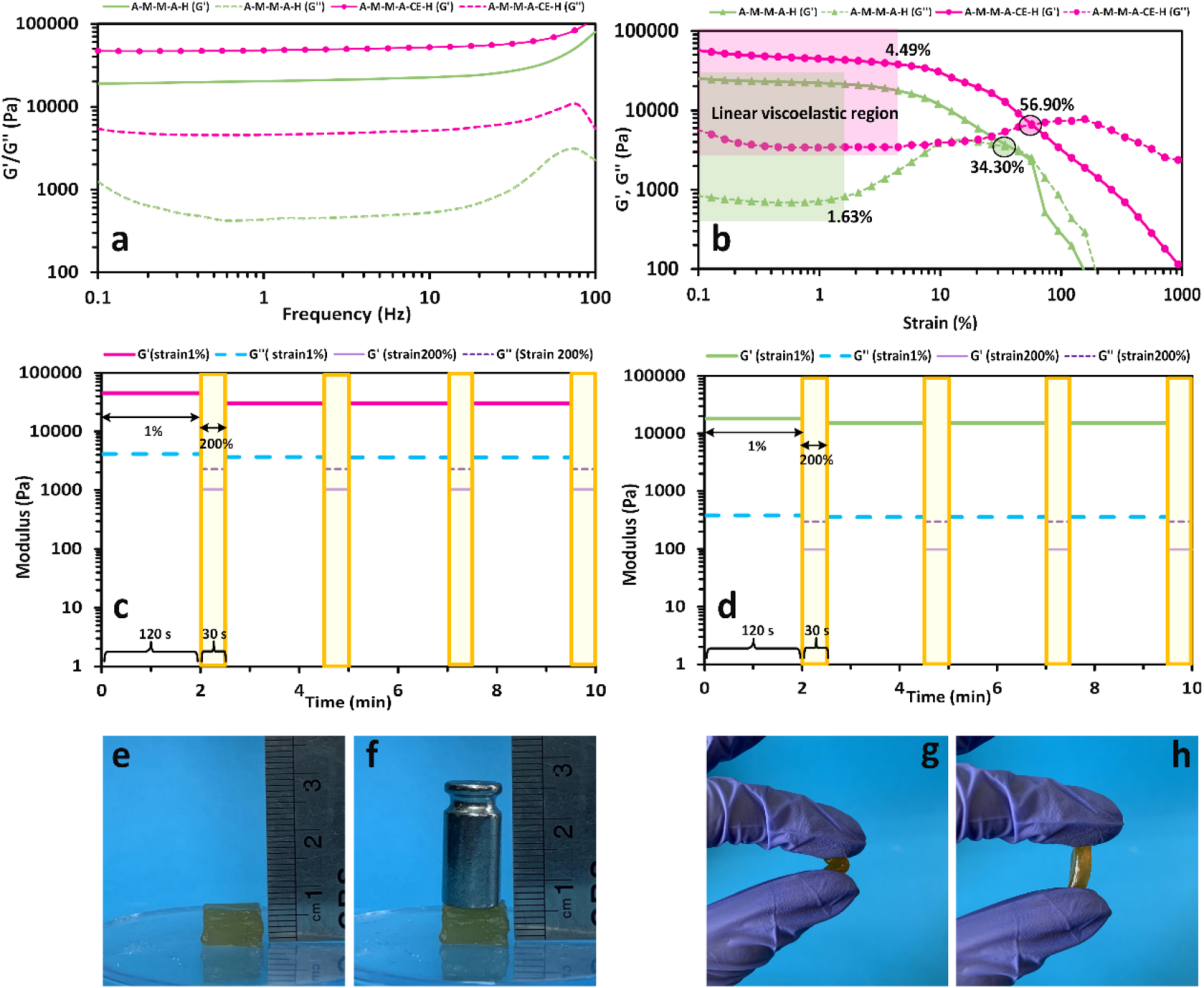
Rheological tests used for hydrogel characterization. (a) *G*′ and *G*″ as a function of frequency sweep test, (b) *G*′ and *G*″ as a function of strain sweep test, (c) changes of *G*′ and *G*″ in alternating strains (1% and 200%) with a duration of 120 and 30 s for A-M-M-A-CE-H at 37 °C, (d) changes of *G*′ and *G*″ in alternating strains (1% and 200%) with a duration of 120 and 30 s for A-M-M-A-H at 37 °C, (e) A-M-M-A-CE-H before weight loading, (f) A-M-M-A-CE-H after weight loading, (g) A-M-M-A-CE-H under finger compression, and (h) A-M-M-A-CE-H after compression.

The mechanical integrity and viscoelastic response of the A-M-M-A-H and A-M-M-A-CE-H hydrogels were evaluated through frequency, strain, and step-strain sweeps (Fig. 7a–h). As shown in Fig. 7a, the frequency sweep tests revealed that *G*′ consistently remained higher than *G*″ for both hydrogels across the entire frequency range (0.1–100 Hz) at 1% strain, indicating a dominant solid-like behavior. The *G*′ values were nearly frequency-independent, confirming the formation of a stable and well-interconnected 3D network. Notably, A-M-M-A-CE-H exhibited a higher elastic modulus, with a value of approximately 46 833 Pa, which is about 2.5-fold higher than that of A-M-M-A-H (∼18 850 Pa). This rheological behavior was consistent with the SEM observations (Fig. 4), in which the CE-containing hydrogel exhibited a more compact, denser microstructure than the control sample. Furthermore, the lower ESR observed (Fig. 6b) for A-M-M-A-CE-H was consistent with its higher elastic modulus, reflecting a tighter, more restricted network structure.

To determine the LVR, strain sweep tests were performed. As shown in Fig. 7b, both hydrogels exhibited a clear LVR, where *G*′ and *G*″ were independent of strain, and *G*′ remained higher than *G*″. This behavior was observed up to approximately 1.63% strain for A-M-M-A-H and 4.49% for A-M-M-A-CE-H, indicating a slightly broader LVR in the presence of CE, beyond their critical strains around 34.30% for A-M-M-A-H and 56.90% for A-M-M-A-CE-H. Beyond the critical strain, a pronounced reduction in *G*′ was observed, reflecting the breakdown of the interconnected network structure. The crossover between *G*′ and *G*″ marked the onset of the gel-to-sol transition. Further increases in strain caused *G*″ to exceed *G*′, indicating a shift from elastic-dominated behavior to viscous flow and revealing the liquid-like characteristics of the hydrogels under large deformations. Interestingly, the A-M-M-A-CE-H group exhibited a higher *G*′ compared to the CE-free sample. This enhancement likely stemmed from additional hydrogen bonding and physical entanglements provided by the phytochemical constituents of the extract, which reinforce the ACSG-based matrix.

The structural resilience, self-recovery efficiency, and thixotropic behavior of the developed hydrogels were studied using step-strain sweep tests, which were performed by alternating strain between 1% and 200% at 120 s and 30 s intervals, respectively (Fig. 7c and d). At 1% strain, which lies within the LVR, the A-M-M-A-CE-H hydrogel showed significantly higher *G*′ values (*G*′ = 45 030 Pa) compared to the A-M-M-A-H sample (*G*′ = 18 600 Pa), indicating a stronger network structure in the presence of the modifying agent. When the strain increased to 200%, the hydrogel network collapsed completely, and the relationship changed to *G*′ < *G*″, confirming the disruption of physical interactions within the hydrogel matrix. Remarkably, upon returning the strain to 1% LVR, an almost rapid structural recovery of the hydrogel network was observed. Within this context, the A-M-M-A-CE-H hydrogel exhibited rapid recovery of its viscoelastic network. The recovery ratio was determined as the ratio of the recovered storage modulus after returning to low strain to the initial storage modulus before large deformation. Based on this calculation, the hydrogel restored approximately 90% of its initial *G*′ within seconds and maintained this recovery behavior over repeated cycles. In comparison, the unmodified A-M-M-A-H hydrogel showed lower recovery efficiency and a slight progressive decline in network stiffness. Images of A-M-M-A-CE-H subjected to weight loading and manual squeezing between fingers are presented in Fig. 7e–h.

This rapid and reversible recovery was likely associated with the dynamic and reversible nature of the ionic crosslinks mediated by Mg^2+^ and CE ions. Furthermore, synergistic physical interactions, such as hydrogen bonding and ionic interaction, which are enhanced in the presence of CE, played a crucial role in improving the self-recovery behavior and maintaining the structural integrity of the hydrogel.

For soft materials exposed to dynamic mechanical conditions, efficient energy dissipation and structural recovery after deformation are desirable features. Designing materials with enhanced elastic response and self-recovery ability under cyclic loading can improve their durability and mechanical performance during repeated deformation. Both A-M-M-A-H and A-M-M-A-CE-H hydrogels demonstrate rapid and repeatable modulus recovery after strain removal, confirming their robust thixotropic behavior and shear-responsive network.

### 3.6. Antibacterial activity and infection control potential

Although antibacterial inorganic agents, including metal and metal oxide nanoparticles, have been incorporated into biomaterials to prevent bacterial growth,^26,54^ naturally derived bioactive compounds have recently attracted considerable attention as potentially biocompatible and sustainable alternatives.^55–57^

**Fig. 8:**
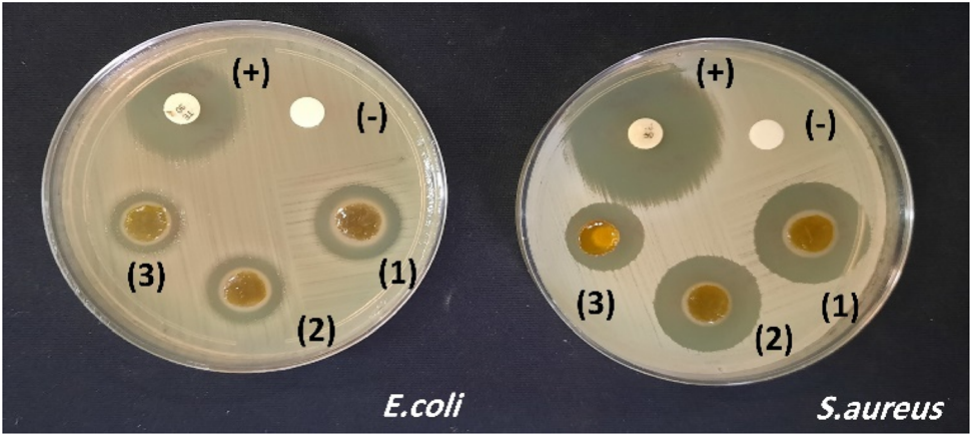
Antibacterial activity of A-M-M-A-CE-H evaluated by inhibition zones against *E. coli* (15.33 ± 1.53 mm) and *S. aureus* (17.67 ± 2.52 mm). Data are presented as mean ± SD (*n* = 3). Whatman paper was used as a negative control (−), and tetracycline as a positive control (+).

To evaluate the antibacterial activity of the developed hydrogels, *S. aureus* and *E. coli* were selected as representative Gram-positive and Gram-negative bacterial strains, respectively. Tetracycline was used as a positive control, and Whatman paper was used as a negative control. Representative images of inhibition zones are shown in Fig. 8. The A-M-M-A-CE-H hydrogel exhibited antibacterial activity against both bacterial strains, with net inhibition zone diameters of 8.33 ± 1.53 mm for *E. coli* and 10.67 ± 2.52 mm for *S. aureus*. The reported inhibition zones were calculated after subtracting the intrinsic hydrogel diameter (7 mm) from the measured inhibition zone diameter. As expected, no inhibition was observed for the negative control, whereas the positive control exhibited higher antibacterial activity, with net inhibition zones of 15 and 25 mm for *E. coli* and *S. aureus*, respectively, after correction for the tetracycline disc diameter. The antibacterial activity observed for A-M-M-A-CE-H may be associated with the combined effects of *chamomile*-derived bioactive constituents, particularly phenolic compounds and flavonoids, and Mg^2+^ ions. The higher susceptibility of *S. aureus* compared with *E. coli* may be related to differences in cell wall structure, as Gram-negative bacteria possess an outer membrane that can limit the penetration of certain bioactive compounds.^58^

### 3.7. Evaluation of cell viability

**Fig. 9:**
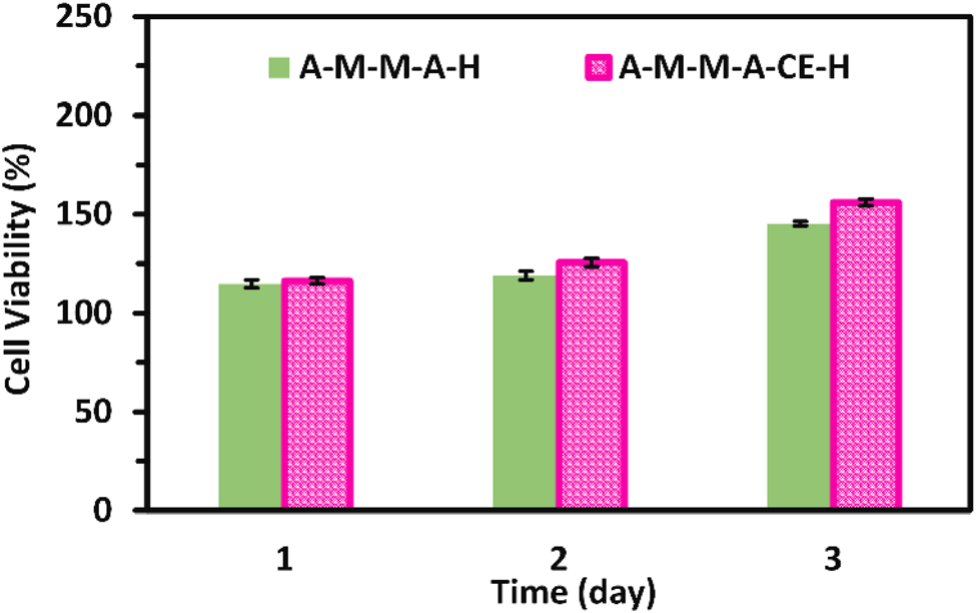
*In vitro* cytocompatibility of A-M-M-A-H and A-M-M-A-CE-H hydrogels evaluated using the MTT assay. Cell viability of H9c2 cells after 1, 2, and 3 days is shown. Data are presented as mean ± SD (*n* = 3); *p* < 0.005.

Cell viability of H9c2 cardiomyoblasts was evaluated to assess the preliminary cytocompatibility of the developed hydrogels. As shown in Fig. 9, both A-M-M-A-H and A-M-M-A-CE-H supported high levels of cellular metabolic activity over 72 h. The viability of cells cultured with A-M-M-A-H increased from 114.63 ± 2.03% at 24 h to 145.21 ± 1.99% at 72 h, while A-M-M-A-CE-H showed an increase from 116.22 ± 1.64% to 156.02 ± 1.83% over the same period. Statistical analysis confirmed that all values were significantly higher than those of the control group (*p* < 0.005).

The observed cell viabilities above 100% suggested that both hydrogels not only exhibit non-cytotoxic behavior but may also enhance cell metabolic activity under *in vitro* conditions. The slightly higher viability observed for A-M-M-A-CE-H may be attributed to bioactive phytochemicals in the CE, including flavonoids and polyphenols, which have been reported to promote cell survival and metabolic activity. In addition, the improved EC of this hydrogel may contribute to a more physiologically relevant environment for electrically active cells such as H9c2. Based on established cytotoxicity classifications,^30^ all samples fall within the non-cytotoxic range (>90%), indicating acceptable cytocompatibility of the developed hydrogels. These findings provide preliminary information regarding the cytocompatibility of the developed hydrogels under *in vitro* conditions. However, further comprehensive biological evaluations, including functional cellular assays, long-term *in vitro* studies, and *in vivo* investigations, are necessary to better understand the biological performance of these materials.

## 4. Conclusions

In this study, a conductive dual-network hydrogel based on ACSG was successfully developed by integrating a chemically crosslinked polyacrylamide network with Mg^2+^-mediated physical interactions. The incorporation of CE was associated with noticeable changes in the hydrogel microstructure and multifunctional performance. The results demonstrated that CE incorporation led to a more compact microstructure with reduced pore size, which was consistent with the observed decrease in swelling ratio. Rheological analysis revealed improved viscoelastic properties, including a higher *G*′, an extended LVR, and enhanced self-recovery behavior under cyclic deformation. The enhanced viscoelastic performance is likely associated with the synergistic effect of inorganic ions and polyphenolic compounds present in CE, which promoted additional ionic and hydrogen-bonding interactions with the polymer matrix. In addition, the hydrogels exhibited improved ionic conductivity in both freshly prepared and equilibrium-swollen states, primarily due to the presence of mobile ionic species introduced by the CE. The developed hydrogels also exhibited antibacterial activity against *S. aureus* and *E. coli* while maintaining high cell viability in MTT assays, confirming their good *in vitro* cytocompatibility. These findings suggest that combining a dual-network architecture with the incorporation of natural bioactive extracts is an effective strategy for tailoring the structural, mechanical, electrical, and biological properties of polysaccharide-based conductive hydrogels. Unlike our previous studies, which focused on improving conductivity and structural performance through conductive nanomaterials or employing plant extracts mainly as conductive additives, the present work investigates the potential dual role of a plant-derived extract in modulating both ionic conductivity and the physicochemical characteristics of a dual-network hydrogel system. Overall, this work demonstrates the potential of ACSG-based dual-network hydrogels modified with CE as multifunctional biomaterials with tunable physicochemical and biological performance. Further studies, including long-term *in vitro* evaluation and *in vivo* investigations, are required to fully assess their applicability in soft tissue engineering, particularly in dynamically loaded biological environments.

## 5. Limitations and future perspectives

Despite the promising physicochemical, rheological, and biological characteristics observed in this study, several limitations should be acknowledged. First, the conductivity of the hydrogels was evaluated using the direct current (DC) resistance method, which provides a rapid estimation of bulk ionic conductivity but does not distinguish between bulk, interfacial, and polarization-related resistive contributions. Therefore, future studies should employ electrochemical impedance spectroscopy (EIS) to obtain a more comprehensive understanding of ion transport behavior within the hydrogel network.

Second, the antibacterial activity was assessed solely by the agar disk diffusion method. While this assay is useful for evaluating the antibacterial effects of diffusible compounds released from the hydrogel, it does not provide information regarding contact-killing mechanisms or long-term antibacterial performance. Accordingly, future investigations should include quantitative bacterial viability assays and prolonged antibacterial evaluations better to characterize the antimicrobial functionality of the developed system.

In addition, the current biological assessment was limited to short-term H9c2 cell viability and antibacterial testing. Although the results indicate good *in vitro* cytocompatibility, further biological validation is required before any biomedical application can be established. Future studies should include analyses of cell adhesion and morphology, live/dead staining, evaluation of the inflammatory response, long-term degradation studies, and more advanced investigations relevant to electroactive tissues. Furthermore, *in vivo* experiments will be necessary to comprehensively evaluate the safety, functionality, and translational potential of the developed hydrogel.

Finally, while the present results suggest that CE may influence the physicochemical characteristics of the hydrogel network, the exact molecular mechanisms underlying these effects require further investigation using complementary characterization techniques and more detailed structure–property analyses.

## Author contributions

Mona Baniasadi: writing – review and editing, writing – original draft, visualization, validation, methodology, investigation, formal analysis, data curation, conceptualization. Mahsa Baghban Salehi: writing – review and editing, validation, supervision, project administration, methodology, investigation, funding acquisition, data curation, conceptualization. Hossein Baniasadi: writing – review and editing, validation, supervision, project administration, investigation, conceptualization.

## Conflicts of interest

There are no conflicts to declare.

## Supplementary Material

RA-OLF-D6RA05964C-s001

## Data Availability

The data that support the findings of this study are available from the corresponding authors upon reasonable request. Supplementary information (SI) is available. See DOI: https://doi.org/10.1039/d6ra05964c.

## References

[cit1] Mahmoudi M., Dargahi M., Mashayekhan S., Jahanmardi R., Baniasadi H. (2025). Int. J. Biol. Macromol..

[cit2] Baniasadi H. (2025). Adv. Colloid Interface Sci..

[cit3] Baniasadi H., Abidnejad R., Fazeli M., Lipponen J., Niskanen J., Kontturi E., Seppälä J., Rojas O. J. (2024). Adv. Colloid Interface Sci..

[cit4] Kaleshtari A. H., Hasani M., Farjaminejad S., Farjaminejad R., Foroozandeh A., Abdouss M., Hasanzadeh M. (2026). RSC Adv..

[cit5] Oktay B., Akkad H., Ahlatcıoğlu Özerol E. (2026). J. Biomater. Sci., Polym. Ed..

[cit6] Stark B. L., Perez J. L., Borrego E. A., Poon W., Natividad-Diaz S. L. (2026). ACS Omega.

[cit7] Baig M. M., Khan S. A., Ahmad H., Liang J., Zhu G., Pang H., Zhang Y. (2024). FlexMat.

[cit8] Holme S., Richardson S. M., Bella J., Pinali C. (2025). Int. J. Mol. Sci..

[cit9] Han M., Jin H., Zhang J., Zhang C., Yang W., Sun D., Huang W. (2026). RSC Adv..

[cit10] Kim H., Narayanan K. B., Kumar V., Han S. S., Bhaskar R. (2026). Biomater. Adv..

[cit11] Hou R., Xie Y., Song R., Bao J., Shi Z., Xiong C., Yang Q. (2024). Cellulose.

[cit12] Liu J., Shen Y., Duan K., He X., Wang R., Chen Y., Li R., Sun J., Qiu X., Chen T., Wang J., Wang H. (2025). J. Mech. Behav. Biomed. Mater..

[cit13] Li C., Chen C., Shi J., Han W., Fu Q., Yang L. (2026). Sci. Adv..

[cit14] Zhou C., Wu T., Xie X., Song G., Ma X., Mu Q., Huang Z., Liu X., Sun C., Xu W. (2022). Eur. Polym. J..

[cit15] Salamat Q., Moradi R., Nadizadeh Z., Kavehpour P., Soylak M., Asimov A., Zillur Rahman M., Kovářík T., Babuška V., Deshmukh K. (2026). Bioact. Mater..

[cit16] Backes E. H., Pinto L. A., Gomes Neto J. F., Lima T. P. L., Granja P. L., Pessan L. A., Porcionatto M. A. (2026). ACS Omega.

[cit17] Simayi R., Wang J., Sawut A., Cheng L. (2026). J. Mech. Behav. Biomed. Mater..

[cit18] Jin Q., Jiang M.-Y., Wang L.-L., Lin S.-Q., Li T., Fu Z.-X., Zhang L., Wu S.-F., Sun Y. (2026). ACS Polym. Au.

[cit19] Liu B., Jiang Y., Tian Y., Li T., Zhang D. (2025). ACS Omega.

[cit20] Zhang J., Liu Z., Sun J., Yao Z., Lu H. (2025). Carbohydr. Polym..

[cit21] Chen H., Luo Z., Chen Q., Chang Y., Zheng J. (2026). Prog. Polym. Sci..

[cit22] Wu K., Liu A., Chen Y., Chen B., Huang Q., Guo W. (2025). Mater. Today Commun..

[cit23] Ryu J., McClements D. J. (2025). Food Hydrocolloids.

[cit24] Zhang B., Zhang M., Jiang C., Yan W., Pan Y., Meng F. (2026). Colloids Surf., B.

[cit25] Polez R. T., Kimiaei E., Madani Z., Österberg M., Baniasadi H. (2024). Int. J. Biol. Macromol..

[cit26] Baniasadi M., Baghban Salehi M., Baniasadi H. (2025). Int. J. Biol. Macromol..

[cit27] Ebrahimi F., Tirgarian B., Farmani J. (2026). LWT–Food Sci. Technol..

[cit28] Sekhavatizadeh S. S., Maftoonazad N., Motashafi H., Nikkhah M., Asnaashari M. (2026). Biotechnol. Rep..

[cit29] Koocheki A., Mortazavi S. A., Shahidi F., Razavi S. M. A., Taherian A. R. (2009). J. Food Eng..

[cit30] Firouz M. M., Baghban Salehi M., Baniasadi M., Mokhtarani B. (2025). Int. J. Biol. Macromol..

[cit31] Zabihi S., Salehi M., Zamani S., Molzemi S., Rahmati M., Abbaszadeh-Goudarzi G. (2025). Int. J. Biol. Macromol..

[cit32] Moshfeghi T., Najmoddin N., Arkan E., Hosseinzadeh L. (2024). Int. J. Biol. Macromol..

[cit33] Jamroży M., Głąb M., Kudłacik-Kramarczyk S., Drabczyk A., Gajda P., Tyliszczak B. (2022). Materials.

[cit34] Harighi H., Baghban Salehi M., Taghikhani V., Mirzaei M. (2024). J. Mol. Liq..

[cit35] Shan J., Jiao W. (2026). RSC Adv..

[cit36] Baniasadi M., Baniasadi H., Azimi R., Khosravi Dehaghi N. (2020). Polym. Eng. Sci..

[cit37] Krysa M., Szymańska-Chargot M., Zdunek A. (2022). Food Chem..

[cit38] Nayak P., Ismayil, Sudhakar Y. N., Shetty S. K. (2024). RSC Adv..

[cit39] Jayaramudu T., Ko H.-U., Kim H. C., Kim J. W., Kim J. (2019). Materials.

[cit40] Kang J., Su X., Zhou P., Yu K., Du Z., Shi D., Yu Y., Lyu X., Zou Z. (2026). Adv. Mater..

[cit41] Lu Y., Zhang R., Gao Y., Liu G., Mao L. (2026). Food Hydrocolloids.

[cit42] Ciarleglio G., Clarizia V., Toto E., Santonicola M. G. (2025). Gels.

[cit43] Revathi D., Panda S., Deshmukh K., Khotele N., Murthy V. R. K., Pasha S. K. K. (2025). Polym. Test..

[cit44] Murphy C. M., O'Brien F. J. (2010). Cell Adhes. Migr..

[cit45] Hajiahmadi S., Baniasadi M., Baghban Salehi M., Dadvand Koohi A. (2026). J. Ind. Eng. Chem..

[cit46] Khademi F., Salehi M. B., Mortaheb H. R., Nozaeim A. A., Ahmadi S. H. (2024). J. Polym. Environ..

[cit47] Harighi H., Kia A. K., Salehi M. B., Mortaheb H. R. (2026). J. Environ. Chem. Eng..

[cit48] Najarbashi N., Baghban Salehi M., Saghandali F., Mokhtarani B., Mirzaei M., Sharifi A. (2024). Fuel.

[cit49] Etemadi Moghaddam H., Baghban Salehi M. (2024). Polym. Adv. Technol..

[cit50] Du Z. H., Wang Q. H., Xiao A. Q., Zhou P. P., Lyu X. L., Zou Z. G. (2026). Chin. J. Polym. Sci..

[cit51] Lee M., Park J., Choe G., Lee S., Kang B. G., Jun J. H., Shin Y., Kim M. C., Kim Y. S., Ahn Y., Lee J. Y. (2023). ACS Nano.

[cit52] Casella A., Panitch A., Leach J. K. (2023). J. Biomed. Mater. Res., Part A.

[cit53] Tohidi H., Maleki-Jirsaraei N., Simchi A., Mohandes F., Emami Z., Fassina L., Naro F., Conti B., Barbagallo F. (2022). Materials.

[cit54] Abu-Gharbia M. A., El-Sayed M. F., Salem J. M., Abd-Elsamei W. M., Al-Arabi G. (2025). Sci. Rep..

[cit55] Ul-Islam M., Habis F., Fatima A., Al Saidi A. K., Shehzad A., Koofan F., Almashli F., Kashoob A. A. A., Ullah M. W., Khan S., El Hariri El Nokab M., Ali M., Sebakhy K. O. (2026). Mater. Adv..

[cit56] Nabilah R., Sin N. M., Rosli N. I., Kumar M., Hilles A. R., Shaik N., Aramwit P., Mahmood S. (2025). ACS Omega.

[cit57] Shahzad S., Ghouse G., Zubair M., Yaqoob A., Mujahid M., Ahmed F., Rauf Z., Ullah A. (2025). RSC Adv..

[cit58] Moghaddam H. E., Salehi M. B. (2026). Mater. Today Commun..

