# Supplementary material for: A conductive and thixotropic dual-network hydrogel based on Alyssum campestre seed gum and Matricaria chamomilla L. extract
Source: RSC Adv. 2026 Aug 12;16(40):47045–56. doi: 10.1039/d6ra05964c (PMC13462857; doi:10.1039/d6ra05964c)
Supplement: Supplementary file 1 [file RA-016-D6RA05964C-s001.pdf]

Supplementary Information (SI) for RSC Advances.
This journal is © The Royal Society of Chemistry 2026

A Conductive and Thixotropic Dual-Network Hydrogel Based on *Alyssum campestre* Seed Gum and *Matricaria chamomilla* L. Extract

Mona Baniasadi[a], Mahsa Baghban Salehi*[a], Hossein Baniasadi†[b]

Table S1. Detailed electrical conductivity measurements of the hydrogel samples

	L (cm)	S (cm^2)	R (ohm)	EC (S/cm)
A-M-M-A-H				
Replicate 1	1	1	5560	1.80E-04
Replicate 2	1	1	1400	7.14E-04
Replicate 3	1	1	2280	4.39E-04
Mean			**4.44E-04±2.67E-04**	
A-M-M-A-CE-H				
Replicate 1	1	1	172	5.81E-03
Replicate 2	1	1	205	4.88E-03
Replicate 3	1	1	234	4.27E-03
Mean			**4.99E-03±7.76E-04**	
A-M-M-A-H (ESR)				
Replicate 1	1	1	833000	1.20E-06
Replicate 2	1	1	274000	3.65E-06
Replicate 3	1	1	164000	6.10E-06
Mean			**3.65E-06±2.45E-06**	
A-M-M-A-CE-H (ESR)				
Replicate 1	1	1	11500	8.70E-05
Replicate 2	1	1	14100	7.09E-05
Replicate 3	1	1	18200	5.49E-05
Mean			**7.09E-05±1.60E-05**	

[a] *Department of Chemical Engineering, Chemistry and Chemical Engineering Research Center of Iran, Tehran, Iran*
[b] *Polymer Synthesis Technology, School of Chemical Engineering, Aalto University, Espoo, Finland.*
* Corresponding author: m.bsalehi@ccerci.ac.ir, mahsa.bsalehi@gmail.com
† Corresponding author: hossein.baniasadi@aalto.fi
